# Exploring viral respiratory coinfections: Shedding light on pathogen interactions

**DOI:** 10.1371/journal.ppat.1012556

**Published:** 2024-09-24

**Authors:** Kylian Trepat, Aurélien Gibeaud, Sophie Trouillet-Assant, Olivier Terrier

**Affiliations:** 1 CIRI, Centre International de Recherche en Infectiologie (Team VirPath), Inserm U1111, Université Claude Bernard Lyon 1, CNRS UMR5308, ENS de Lyon, Lyon, France; 2 Joint Research Unit Hospices Civils de Lyon-BioMérieux, Hospices Civils de Lyon, Lyon Sud Hospital, Pierre-Bénite, France; Mount Sinai School of Medicine, UNITED STATES OF AMERICA

Respiratory infections pose a significant global health burden, being one of the leading causes of illness and death worldwide [1,2]. In 2019, there were an estimated 17.2 billion cases of upper respiratory infections, accounting for over 40% of all illnesses globally [3]. The primary viruses responsible for these infections include respiratory syncytial viruses (RSVs), influenza viruses, and human rhinoviruses (hRVs), all of which can cause a range of symptoms that themselves vary from mild to severe [4]. Additionally, severe acute respiratory syndrome coronavirus 2 (SARS-CoV-2), which emerged in 2020, has significantly impacted public health and now cocirculates with other respiratory viruses. Several respiratory viruses can infect the same individual during a respiratory infection, as evidenced by an increased documentation of viral codetection. Multiplex panels, which detect multiple viral pathogens at once, have revealed that respiratory coinfections are more common than previously thought; these are found in 3% to 26% of patients hospitalised for respiratory infection [5,6]. Coinfections are particularly prevalent in children, the elderly, and immunocompromised individuals, but the impact on the severity of infection or hospitalisation risk is still debated [7,8]. Consequently, comprehensive research into the interactions among various respiratory viruses is crucial for advancing our knowledge and management of these infections.

This Pearl will cover our current understanding of the interactions between respiratory viruses at the host cell level. It will also discuss the challenges in this field, such as the limitations of experimental models, the complexities of analysing epidemiological data, and the need for new approaches to understand deeply these complex coinfections.

## What are we talking about? The different coinfection scenarios

**Fig 1:**
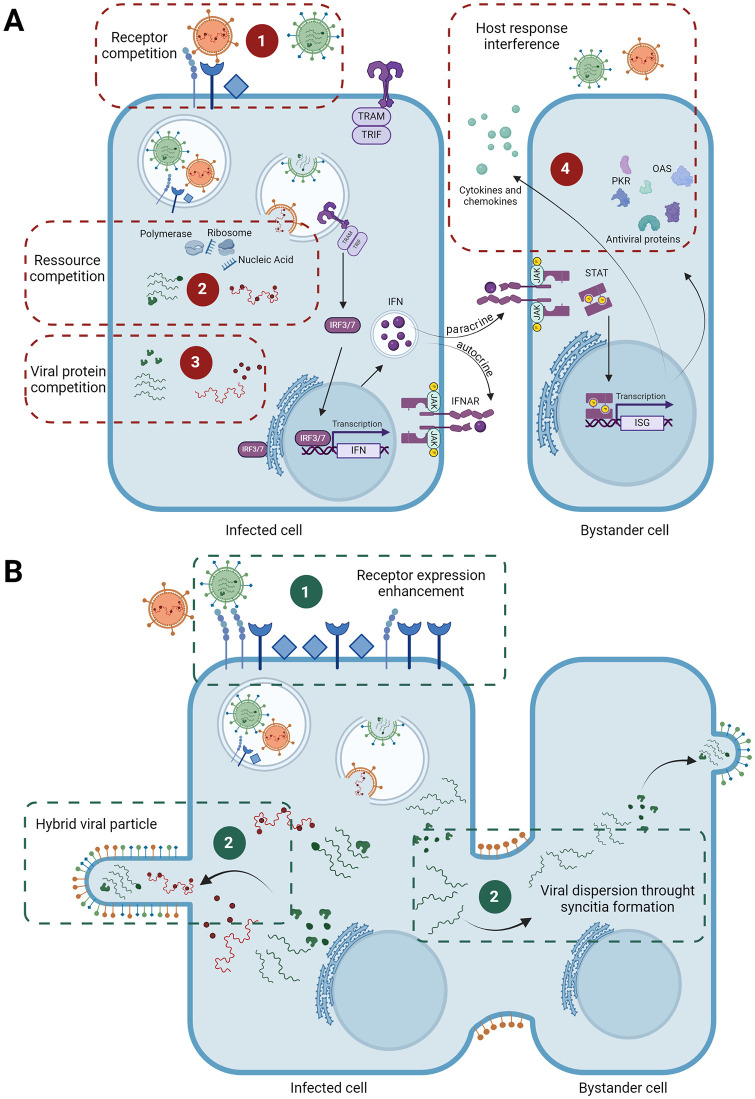
Viral interactions during an infectious cycle. **(A)** The mechanism implicated in negative interactions during a viral coinfection that can be defined as **1-** Competition for receptors; **2-** Competition for cellular resources; **3-** Competition by viral proteins; **4-** Host response induced by one virus. **(B)** The mechanism implicated in positive interaction during a viral coinfection that can be defined as **1-** Increased receptor expression; **2-** Formation of hybrid viral particles; **3-** Syncytia formation facilitating viral spread and increase the replication of another virus. Created with Biorender.

We can distinguish 3 types of interactions between viruses: homologous interactions that involve at least 2 viruses belonging to the same family (e.g., RSV and hMPV), heterotypic that involve 2 viruses belonging to the same species (e.g., influenza virus H1N1 and H3N2), and heterologous that involve 2 viruses belonging to different families (e.g., SARS-CoV-2 and influenza virus). It is essential to understand that viral coinfections can occur simultaneously or sequentially. Interaction between viruses can be qualified as negative or positive according to whether infectivity and/or viral replication are hindered or enhanced. The known mechanisms underlying negative interactions involve different stages of infection are competition for receptors [9,10], competition for cellular resources [11–13], competition through viral proteins [14], which can lead to superinfection exclusion (SIE) [15], and interference mediated by the host response [16] (Fig 1A). The latter plays a crucial role in viral interactions, particularly through the type I/III interferon (IFN) response, which is the most extensively studied mechanism of viral interference. In contrast, data regarding other types of negative interactions are scarce. IFNs, induced by pathogen recognition receptors such as Toll-like receptors (TLRs; i.e., TLR3 recognises double-stranded RNA, TLR7/8 recognises single-stranded RNA, and TLR9 recognises DNA), activate a signalling pathway involving transcription factor as interferon regulatory factor 9 upon binding to their receptors. This leads to the transcription of IFN-stimulated genes (ISGs), which encode hundreds of antiviral proteins such as double-stranded RNA-activated protein kinase and 2′-5′-Oligoadenylate Synthetase (OAS), inhibiting translation and degrading RNA, respectively, 2 proteins involved in negative interactions [17,18]. Very few studies have described viral positive interactions, but receptor expression enhancement [19–21], formation of hybrid viral particles that combine elements from 2 distinct viruses that have a wider receptor tropism [22] and the formation of syncytia that enhances viral dispersion and increased viral replication [23] (Fig 1B).

## Negative interactions: Viral interference but not only…

Although several studies on viral interactions address homologous, heterotypic, and heterologous interactions, most epidemiological studies that establish positive and negative interactions, as well as those associating severity and mortality risks, mainly focus on heterologous interactions between respiratory viruses from different families. Moreover, epidemiological studies may lack the power to identify interactions between viruses that are detected in lower proportions, such as human metapneumovirus (hMPV), even though interaction mechanisms are identified in vitro [24,25]. Additionally, some studies mainly examine the role of the host response, particularly the type I/III IFN response, during heterologous viral interactions using both in vitro and in vivo models. Many studies highlight hRV as a key player involved in negative interactions [16,24,26–29], notably by inducing the up-regulation of genes specifically associated with the inhibition of influenza virus (i.e., mucin gene *MUC1*, *MUC4*, *MUC5AC*, *MUC5B*, and MX dynamin like GTPase 1 [*MX1*]) [29–31]. This immune cellular response induced by hRV has also been described to offer a protective effect up to 14 days postinfection against influenza A virus (IAV; H3N2 subtype) [32]. Given the temperature-restricted properties of hRV, the question may arise as to whether these interactions can take place in vivo. Other studies have also underlined negative interactions between IAV and SARS-CoV-2, notably by comparing the induced host responses following single or coinfection between these 2 viruses [33–35]. This mutual negative interaction seems to be mediated by a virus-specific host response as illustrated by the secretion of C-C motif chemokine ligand (CCL)-5/C-X-C motif chemokine ligand (CXCL)-9 and the rapid and huge type I IFN response leading to the expression of ISGs (OAS1 and CXCL10) by IAV that inhibit SARS-CoV-2 [33–36]. Conversely, SARS-CoV-2 infection has been described to induce MX1 expression and enhance immune cell recruitment, as well as a pro-inflammatory profile/environment through an increase of associated chemokines (CCL2, CCL3, CCL4, CCL5, CCL7, and CXCL10) or cytokines (interleukin (IL)-1, IL-18, and IL-6) impeding IAV replication [33–37]. Consequently, the mechanism leading to negative interaction varies according to which virus infects first. Given the central role of the host response in establishing negative interactions, it is crucial to allow sufficient time between the first and second viral infection to permit the development of the immune response after the initial infection. Therefore, the choice of the study model and the timing of sequential infections are essential for dissecting the mechanisms.

Concerning RSV, notable for its substantial impact on public health, studies have shown that the type I IFN response induced by RSV can alter coinfection with IAV and hMPV [25,38]. However, studies show that IAV induces an IFN response leading to IFN-induced protein with tetratricopeptide repeats (IFIT) expression resulting in an impairment of secondary infection by RSV [39]. This inhibition of RSV replication and infection by IAV can be explained by increased natural killer T cells and earlier induction of CD4+ cells associated with earlier viral clearance induced by primary IAV infection [40].

Overall, these studies demonstrate the IFN-dependent viral interference mechanisms at play between hRV, SARS-CoV-2, RSV, IAV, and hMPV. They also highlight the importance of experimental design regarding infection sequence, or lack thereof, and the relevance of the model used that can lead to numerous variabilities in the interactions found. Furthermore, while studies have highlighted the significance of the type I IFN response in viral interference, evidence of IFN-independent mechanisms exist and need to be further explored [11,12].

## Positive interactions: When one virus paves the way for another

Positive interactions are mostly evidenced by epidemiological studies, where the simultaneous circulation of viruses can lead to preferential associations as evidenced with adenovirus and parainfluenza virus or RSV and hMPV [24]. Despite that the type I/III IFN plays a crucial role in viral negative interaction, this immune response can also be implicated in viral positive interaction. An increase in angiotensin converting enzyme 2 (ACE2) expression, identified as an ISG [41], has been observed following IAV or RV infection, which leads to increased infectivity and replication of SARS-CoV-2 [19–21]. Nevertheless, Busnadiego and colleagues reported that the overall antiviral action of IFN response counterbalances any proviral effect of ACE2 induction [42]. In addition, SARS-CoV-2 infection would up-regulate α-2,3 sialic acids on the cells surface, explaining the rise in IAV viral load and suggesting a potential reciprocal enhancement between the 2 viruses [21]. Although a relevant type II alveolar organoid model was used, the study did not examine how SARS-CoV-2 infection affects the expression of α2–6 sialic acids, for which human influenza viruses have a greater affinity.

More recently, a study identified a new potential mechanism of virus interaction that could expand the tropism of entry receptors for RSV and IAV. The authors identified the formation of hybrid viral particles containing the genome of both IAV and RSV and bearing on their surface both viral glycoproteins, thus conferring broadened receptor tropism for these viral particles [22]. However, confirmation of these findings is needed.

In addition, an interesting mechanism has been described regarding the coinfection of IAV and parainfluenza virus (PIV), given the ability of PIV to form syncytia via its fusion protein F would enhance the replication of IAV by promoting the dispersion of its genome, reaching the nuclei of the fused cells [23].

These 3 mechanisms represent our current understanding of positive interactions to the best of our knowledge, suggesting complex interactions during coinfection and highlighting the importance of dissecting the mechanisms behind positive interactions.

## Challenges and prospects

Our understanding of respiratory viral coinfections, derived from both in vitro and in vivo experimental methods, still needs to be completed. Most research has concentrated on negative interactions, particularly viral interference. Future studies should aim to explore the less understood aspects of these interactions, such as the role of type III IFN in defending the respiratory epithelium and IFN-independent mechanisms such as competition for receptors. Positive interactions between viruses remain largely understudied.

Investigating respiratory coinfections is particularly challenging due to the diversity of viral models and the specialised tools and expertise required. Developing relevant experimental models, such as reconstituted human epithelium models, is crucial. These models must account for the varying tropism of different viruses, which can significantly influence observations. One technical challenge is incorporating an immune component, possibly by integrating primary immune system cells into these models. Moreover, in epidemiological studies as well as in vitro/in vivo models dissecting coinfection, the role of preexisting immunity induced by previous vaccination or infection is poorly studied. Studying coinfections involves managing numerous experimental parameters (timing, sequence, inoculum, immune cells, etc.), making comprehensive testing difficult. Therefore, combining mathematical modelling approaches is essential to manage this complexity.

Last but not least, our current knowledge is based primarily on experimental approaches and may only partially reflect observations at the population level. To bridge this gap, we must adopt multidisciplinary approaches that examine interactions between respiratory viruses across multiple scales, from codetection in patients to detailed in vitro studies using biologically relevant models.
